# Dementia Risk Due to Traumatic Brain Injury in Subtypes of Dementia in the Welsh Population

**DOI:** 10.1212/WNL.0000000000213866

**Published:** 2025-07-03

**Authors:** Emily Simmonds, Jun Han, George Kirov, David J. Sharp, Thomas H. Massey, Valentina Escott-Price

**Affiliations:** 1 UK Dementia Research Institute, Cardiff University, United Kingdom;; 2 Centre for Neuropsychiatric Genetics and Genomics, Cardiff University, United Kingdom;; 3 Faculty of Medicine, Department of Brain Sciences, Imperial College London, United Kingdom; and; 4 Dementia Research Institute, Imperial College London, United Kingdom.

## Abstract

**Background and Objectives:**

Traumatic brain injury (TBI) can increase vulnerability to neurodegenerative disorders. The association between TBI and dementia has been previously reported, but studies have relied on self-reporting of TBI and often do not appropriately adjust for relevant risk factors or cover enough time to include both individuals at age of highest TBI risk and age of dementia onset. This study uses electronic health records, which include over 20 years of data and 1.7 million individuals with hospital or general practitioner diagnoses of dementia and TBI. Therefore, the aim of this study was to assess the association between TBI and dementia and between TBI and dementia subtypes (Alzheimer disease [AD], vascular dementia [VaD], and unspecified dementia).

**Methods:**

We performed a population-based study using Welsh (UK) electronic health records to estimate effect of TBI on the risk of dementia for individuals aged between 30 and 65 years in 1999 without a previous dementia diagnosis. The long-term risk of dementia after TBI was established using Cox proportional hazard models adjusting for sex, social deprivation, and other comorbidities. The effect of the time between TBI and dementia was investigated with time-stratified analyses. We assessed separately the risks of AD, VaD, and unspecified dementia related to TBI.

**Results:**

Our study investigated 42,974 individuals with dementia (mean diagnosis age of 70 [SD = 10.5]), 10,164 individuals with a history of TBI, and 1,737,480 controls (mean age of 62 [SD = 11.9]). 49% of all individuals were female. TBI was associated with increased risk of dementia (hazard ratio [HR] = 2.32, 95% CI [1.88–2.85], *p* = 3.8 × 10^−15^), with the risk increasing for multiple TBIs (HR = 1.22, 95% CI [1.08–1.38], *p* = 1.8 × 10^−03^). The effect size of association between TBI and dementia was higher in people diagnosed with VaD (HR = 1.71, 95% CI [1.06–2.75], *p* = 0.027) and unspecified dementia (HR = 1.90, 95% CI [1.29–2.80], *p* = 0.0011) compared with the AD group (HR = 1.44, 95% CI [0.84–2.48], *p* = 0.189).

**Discussion:**

Our study confirms that TBI increases dementia risk. We have shown a higher risk of VaD and unspecified dementia in those with a TBI, compared with AD. This study will direct future research into which biological mechanisms drive the association between TBI and dementia.

## Introduction

Traumatic brain injury (TBI) is a significant public health issue that is increasing in global incidence and recognition.^1-5^ TBI is caused by a forceful blow to the head or body, which moves the brain within the skull. TBI can be caused by sports injuries, falls, road traffic accidents, or being hit with an object.^6^ The cause of TBI differs substantially between high-income countries (HICs) and low-middle–income countries (LMICs), with falls being the most common cause in HICs and road traffic accidents often involving pedestrians or motorcyclists being most prevalent in LMICs.^7^ There are likely other causes of TBI, which are widely under-reported, such as intimate partner violence. The increasing population of TBI survivors^8^ and growing incidence of dementia with aging populations have led to the observation that TBI may increase an individual's risk of dementia in subsequent years. However, there remains controversy about the true risk of developing dementia from TBI and what causes this association.

A recent review of 44 studies investigating the relationship between TBI and dementia reported that 56.8% of studies found a positive association between TBI and dementia.^9^ The meta-analysis was limited by heterogeneity in both exposure and outcome reporting and by poor study quality, especially due to the scarcity of large-scale studies with long-term follow-up.^9^ Other limitations in previous studies investigating the link between TBI and dementia include using self-reporting to ascertain TBI^9-11^; absence of adjustment for risk factors in performing multivariate analysis; and widespread deficiencies in the recording of dementia onset.^9,11^ Indeed, the dementia diagnosis rate in England was 62% in 2022,^12^ and after COVID-19, diagnosis waiting periods have increased and may be up to 2 years.^13^ Furthermore, owing to overlap of clinical presentations, the diagnosis of neurodegenerative diseases is challenging.^14,15^

In a critical review of TBI study methodology,^11^ the authors flag an important issue of reverse causality, which refers to the possibility that TBI may have occurred after dementia onset caused by prodromal symptoms. This is most often caused by incorrect measurement of the date of TBI, inaccurate estimates of dementia onset, and insufficient time between TBI and dementia.

Some of the strongest evidence linking TBI and dementia is from Scandinavia. Three independent observational studies in Scandinavian populations showed a long-term risk of dementia after TBI (hazard ratio [HR] = 1.9, 1.24, 1.7).^16-18^ The authors report that dementia risk stratified by time since TBI was higher when TBI occurred at a younger age^17^ and that dementia risk is highest in the first few years after TBI, which may indicate the presence of undiagnosed dementia (reverse causality), and this risk is sustained for more than 30 years after the TBI.^18^ It has also been shown that the risk of dementia increases with an increasing number of TBI events.^17,18^ Furthermore, dementia risk was shown to be higher when considering TBI events compared with traumatic fracture not involving the skull or spine,^17^ and mild TBI showed a weaker association with dementia compared with more severe TBI.^18^ It has been reported that TBI induces neurodegenerative processes, which may be associated with early dementia, for example, chronic traumatic encephalopathy.^19^

There are limited data on the risks of dementia subtypes after TBI. A systematic review^20^ of the association of TBI with Alzheimer disease (AD) onset revealed that 55.5% of patients with TBI may have impaired cognition, from acute post-TBI cognitive deficits to later meeting diagnostic criteria for AD. A review of longitudinal population studies found that TBI increased the risk of AD when studies were pooled.^21^ However, whether TBI is a risk factor of AD remains elusive,^20^ although there is evidence that in individuals with AD, disease onset was 2.5 years earlier if there was a history of TBI.^22^ There is little in the literature defining the association between TBI and vascular dementia (VaD), despite the link between TBI and stroke^23^ and the increased prevalence of vascular injury after TBI.^24^ In a study of around 23,000 individuals, in the 5 years after TBI, patients have approximately double the risk of stroke.^25^ To add to the complexity, a recent report^15^ showed that 17% of individuals with a diagnosis of AD had also been diagnosed with VaD and approximately 20% of people with a diagnosis of VaD had a diagnosis of AD.

Population-based analyses with large sample sizes and long follow-up periods are essential in providing convincing evidence on the association between TBI and dementia risk. Ideally, this would involve prospective studies following cohorts of individuals with and without TBI. However, given that dementia may occur many years after TBI, such studies are extremely expensive in time and resources. Therefore, well-designed retrospective studies that draw on large populations followed over tens of years are likely to be the most practical way in the short term of providing insight into associations between TBI and dementia. In this study, we hypothesized that TBI is associated with an increased risk of dementia and that the risk is higher in people with multiple TBIs. We investigated whether the risk is the same for different subtypes of dementia, specifically VaD, AD, and unspecified dementia, and also subtypes of TBI (i.e., concussion, intracranial injury, and skull fracture).

## Methods

Our study used an observational cohort from Wales using UK National Health Service (NHS) administrative and clinical datasets for primary and secondary care records stored in the Secure Anonymised Information Linkage (SAIL) databank,^26^ containing anonymized individual-level, population-scaled data, sized over 4.5 million persons without any filtering. The SAIL databank was originally a repository of health data and has expanded to include administrative data; therefore, individuals do not enroll into the study, providing a representative sample of the population.

### Standard Protocol Approvals, Registrations, and Patient Consents

Ethical approval to perform this work was provided by the Information Governance Review Panel (IGRP) of SAIL, project number 0998. All data contained in SAIL are managed by the relevant Caldicott Guardian or Data Protection Officer. All methods were performed in accordance with the relevant SAIL guidelines and regulations. The SAIL databank is an anonymized databank that does not handle identifiable data. SAIL acts as data guardians, and therefore, individual informed consent is not required (saildatabank.com/governance/approvals-public-engagement/information-governance/).

### Data Extraction and Inclusion Criteria

We retrieved up to 20 years (from January 1, 1999, to December 31, 2018) of primary care and hospital records. This study included individuals who were alive, living in Wales, and aged between 30 and 65 years and had not received a dementia diagnosis at the beginning of the study (1999).

Dementia cases in this study were defined as individuals with their first dementia diagnosis during the study period in either the hospital datasets or the Welsh Longitudinal General Practitioner (WLGP) dataset, a dataset containing records from Welsh primary care. In both instances, all types of dementia were queried, including nonspecific diagnoses. Unspecified dementia is a general term for dementia that is not linked to a specific disease or diagnosis. Age at onset was defined as the age at first dementia diagnosis. ICD-10 codes and NHS Read codes (Clinical Terms Version 2, CV2) (used for WLGP) for identifying dementia cases are listed in eTable 1. If individuals were recorded as having more than one type of dementia, that is, dementia and AD, they were included in both analyses, but the age at onset was defined as the first recorded diagnosis of any dementia phenotype. Controls were defined as individuals who have never had any dementia diagnosis in all available SAIL records. This resulted in a total of 42,974 dementia cases and 1,737,480 controls. There was no minimum time set between TBI and dementia diagnosis. Individuals with a diagnosis of dementia linked to substance (both alcohol and drug) abuse were excluded from this analysis because this type of dementia is not always a progressive condition and, therefore, may have a different disease mechanism.

Data for TBI diagnosis were extracted from the hospital datasets in SAIL, including the Patient Episode Database for Wales (PEDW), the Outpatients Database for Wales (OPDW), and the Emergency Department Dataset (EDDS). ICD-10 codes and Accident and Emergency codes for TBIs (concussion, intracranial injury, and skull fracture) were used for data extraction from PEDW and OPDW, and EDDS, respectively (eTable 2). Only TBI occurring before the end of the study or dementia diagnosis were included in the study. Multiple TBI diagnoses with the same diagnosis code within 4 weeks were counted as a single TBI event with multiple hospital visits; the 4-week cutoff was based on visual inspection of the data. The diagnosis date for TBI was based on the last TBI event.

Demographic information, including week of birth, sex, and Townsend social deprivation index quintiles, was derived from the Welsh Demographic Services datasets.

History of medical comorbidities was retrieved from the WLGP dataset. We constructed a comorbidity measure (Charlson Comorbidity Index [CCI]) as the number of comorbid disorders per individual, which is frequently used for risk adjustment by health care researchers.^27^ ICD-10 codes^27^ were used to generate CCI and include liver disease, tumor, diabetes, rheumatic disease, congestive heart failure, pulmonary disease, cerebrovascular disease, peptic ulcer disease, hemiplegia and paraplegia, renal disease, and myocardial infarction. In addition, ICD-10 codes and Read codes for psychiatric conditions (including disorders with psychosis, mania, depression, and anxiety) were obtained from the hospital and general practitioner (GP) data (PEDW, OPDW, and WLGP datasets, eTable 3).

### Data Analysis

HRs and the corresponding 95% CIs for the risk of dementia and TBI, as well as the number of TBIs (individuals reporting 1, 2, or 3+ TBIs), were estimated using Cox proportional hazard models. We also looked at the influence of TBI on dementia risk split by the age TBI occurred (<60 or 60+). Data were censored for controls using age at the end of study, that is, age at death, age an individual left Wales, or age on 31.12.2018, whichever came first, and age at dementia diagnosis in cases. We adjusted for sex, Townsend social deprivation and interaction between TBI and sex (TBI*sex), the CCI, and psychiatric disorders (age was already adjusted for in the Cox model).

As secondary analyses, we used Cox models to assess (1) the effect of different types of TBI (concussion, intracranial injury, and skull fracture) on dementia risk; (2) the effect of TBI on dementia risk stratified by the time between the last TBI date and dementia diagnosis (or the end of the study in controls) using the intervals <1, 1–5, and 5+ years; and (3) risk of AD, VaD, and unspecified dementia, excluding those who had TBI within 1 year of diagnosis. These time intervals were chosen to give similar sample sizes in each subgroup. When investigating the association between TBI and VaD, we included a cardio adjustment, which also accounted for the presence of ischemic heart disease and high blood pressure in addition to the CCI, which included diabetes. As sensitivity analysis, owing to the overlap between AD and VaD, we have also analyzed the data where individuals with both diagnoses were excluded.

All analyses were performed in *R* version 4.1.3.^28^ The Bonferroni-corrected significance threshold was set to 0.005 (=0.05/10): association with dementia in the whole sample and in 3 diagnosis subgroups (AD, VaD, unspecified), 3 TBI types, and 3 time intervals between the last TBI date and dementia diagnosis. *p* values are reported without correction for multiple testing, but, where appropriate, we state whether the *p* values would survive correction for multiple testing.

### Data Availability

The datasets supporting the conclusion of this research are accessible through the SAIL platform. Application to view and use these data must be approved by the Information Governance Review Panel. More information is available in the SAIL guidelines at saildatabank.com/data/.

## Results

### Demographic and Clinical Characteristics

The number of individuals alive, living in Wales, and aged between 30 and 65 years in 1999 were N = 1,780,454. Individuals were followed up for 20 years, and whether they experienced a TBI or were diagnosed with dementia was recorded. A total of 42,974 (2.4%) developed dementia, and 10,164 (0.6%) experienced a TBI in the study period. Medical notes were examined between 1990 and 1999, and individuals with any dementia diagnosis during that time were excluded. We also excluded individuals diagnosed with other neurodegenerative disorders not studied here, such as Parkinson disease, multiple sclerosis, Huntington disease, and frontotemporal dementia. The demographics of the study data are given in Table 1.

**Table 1:** Demographic and Clinical Characteristics of the Sample

	Participants with dementia	Participants with no dementia
	N = 42,974 (2.4%)	N = 1,737,480 (97.6%)
Sex (%)		
Male	21,286 (49.5)	886,209 (51.0)
Female	21,688 (50.5)	851,263 (49.0)
Townsend quintiles (%)		
1 least deprived	7,048 (16.4)	337,142 (19.4)
2	10,305 (24.0)	413,676 (23.8)
3	11,702 (27.2)	495,152 (28.5)
4	10,210 (23.8)	351,640 (20.2)
5 most deprived	3,709 (8.6)	139,870 (8.1)
TBI (%)		
No	42,151 (98.1)	1,728,139 (99.5)
Yes	823 (1.9)	9,341 (0.5)
Type of injury (%)		
Intracranial	654 (1.5)	6,329 (0.4)
Skull fracture	137 (0.3)	2,190 (0.1)
Concussion	32 (0.07)	822 (0.05)
Numbers of TBI (%)		
0	42,151 (98.1)	1,728,139 (99.5)
1	615 (1.4)	7,083 (0.4)
2	162 (0.4)	1,824 (0.1)
3+	46 (0.1)	434 (0.02)
Psychiatric disorder (%)		
No	27,156 (63.2)	1,432,614 (82.5)
Yes	15,818 (36.8)	304,866 (17.5)
CCI: number of Charlson comorbidities (excl. HIV and dementia) (%)		
0	18,170 (42.3)	1,284,460 (73.9)
1	8,536 (19.9)	171,250 (9.9)
2	4,567 (10.6)	115,279 (6.6)
3	3,944 (9.2)	71,648 (4.1)
4	3,224 (7.5)	41,171 (2.4)
5+	4,533 (10.5)	53,672 (3.1)
Type of dementia (%)		
AD	17,253 (40.1)	—
VaD	12,626 (29.4)	—
Unspecified	29,580 (68.8)	—
Both AD and VaD^a^	3,318 (7.7)	—

Abbreviations: AD = Alzheimer disease; CCI = Charlson Comorbidity Index; HIV = Human Immunodeficiency Virus; TBI = traumatic brain injury; VaD = vascular dementia.

a Note that some individuals are diagnosed with both AD and vascular dementia.

There was little difference in most demographics presented between cases and controls. Dementia cases had a higher proportion of psychiatric disorders and other Charlson comorbidities compared with controls. The mean age at first diagnosis of dementia was 70.0 years (SD = 10.5), and the mean age at the end of the study/age at death/age an individual left Wales in controls was 61.5 years (SD = 11.9). In the study cohort, 10,164 individuals had at least one hospital contact between January 1, 1999, and December 31, 2018, which resulted in a TBI diagnosis. Dementia cases had a higher proportion of individuals with a previous TBI (1.9%) compared with controls (0.5%) (Table 1).

All types of dementia were included in the cases, with N = 29,580 (68.8%) being unspecified dementia, N = 17,253 (40.1%) being AD, and N = 12,626 (29.4%) being VaD. Some people received multiple diagnoses. The Figure shows the number of dementia subtypes with their overlap. There were 2,095 individuals who received a diagnosis of all 3 dementia subtypes, AD, VaD, and unspecified dementia. Unspecified dementia had the largest sample size with 15,968 individuals receiving only an unspecified dementia diagnosis.

**Figure:**
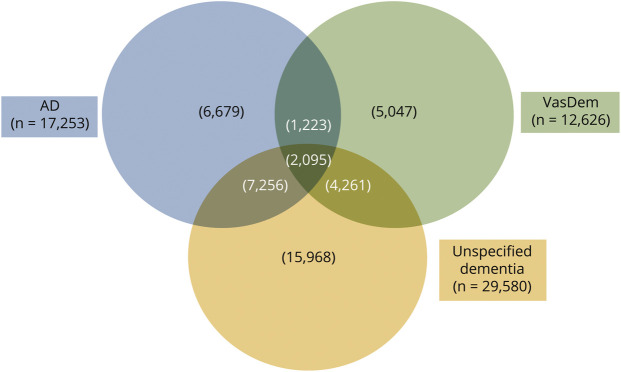
Venn Diagram Showing the Overlap Between Dementia Subtypes in the Whole Sample

### Risk of Dementia Associated With TBI

We found that the risk of dementia in people with a history of TBI was higher (HR = 2.32, 95% CI [1.88–2.85], *p* = 3.8 × 10^−15^) than in those with no history of TBI adjusting for sex, Townsend social deprivation index, the interaction between TBI and sex, and the comorbid adjustment, which further adjusts for history of psychiatric disorders and the CCI. All variables used for adjustments were significant predictors of dementia risk, with psychiatric disorders, other comorbidities, and lower socioeconomic status increasing the risk. The influence of TBI on dementia risk is higher when the individual experienced a TBI at age younger than 60 (HR = 6.4, 95% CI [4.28–9.57], *p* = 1.4e-19) compared with individuals older than 60 experiencing a TBI (HR = 1.62, 95% CI [1.26–2.07], *p* = 1.3e-04).

Among individuals with a history of TBI, most (N = 6,983, 68.7%) had a diagnosis of a significant intracranial injury such as cerebral contusion or laceration, or traumatic intracranial hemorrhage. An additional 22.9% (N = 2,327) of the patients had sustained a skull fracture. Note that only a small proportion (N = 854, 8.4%) had concussion diagnosed. The risk of dementia was greater for intracranial injury and skull fracture (HR = 2.48, [95% CI 1.97–3.14], *p* = 2.5 × 10^−14^; HR = 2.06, 95% CI [1.24–3.43], *p* = 0.0053) compared with concussion (HR = 1.01, 95% CI [0.35–2.98], *p* = 0.981).

For individuals with a history of TBI, the risk of dementia further increased with multiple TBI events (HR = 1.22, 95% CI [1.08–1.38], *p* = 0.0018), although there were only a small number of individuals (N = 2,466) recorded with more than one TBI event.

### Time From the TBI to Dementia

Table 2 provides the effect of TBI on dementia risk, stratified by the time between TBI and dementia diagnosis/end of study. The effect of TBI on dementia risk was significantly higher when TBI occurred within 1 year of dementia diagnosis or the end of the study with HR > 4. The remaining time intervals showed a consistent effect of TBI on dementia risk, with a HR of approximately 2. Note that also the CIs of HRs of the “<1-year” analysis and other time intervals did not overlap. The consistent HRs when the time between TBI and diagnosis is greater than 1 year suggest that the influence of reverse causality can be reduced by excluding TBIs occurring within 1 year of dementia diagnosis. Therefore, all analyses presented from now on show results for the sample excluding TBI within 1 year of dementia diagnosis.

**Table 2:** Multivariate Cox Proportional Hazard Regression Results for the Effect of History of TBI on Risk of Dementia Split by Time Intervals Between the Last TBI and Dementia Diagnosis

Time since TBI	N with dementia and TBI	HR (95% CI)	*p* Value
<1 y	329	4.12 (2.96–5.74)	4.7 × 10^−17^
1–5 y	231	1.76 (1.19–2.62)	0.005
5+ yrs	263	1.95 (1.35–2.80)	0.0003

Abbreviation: TBI = traumatic brain injury.

### Association Between TBI, VaD, and Unspecified Dementia

To explore the association between TBI and dementia subtypes (AD, VaD, and unspecified dementia), we ran Cox proportional hazard models looking specifically at these dementia subtypes; results are presented in Table 3.

**Table 3:** Multivariate Cox Proportional Hazard Regression Results for the Effect of History of TBI on Risk of Dementia in Dementia Subtypes (AD, Vascular Dementia, and Unspecified Dementia), Excluding TBI Occurring Within 1 Year of Diagnosis

Dementia subtype	N with dementia	N with dementia and TBI	HR (95% CI)	*p* Value
AD	17,180	123	1.44 (0.84–2.48)	0.189
AD (excluding VaD)	13,884	92	1.42 (0.76–2.67)	0.273
VaD^a^	12,530	158	1.71 (1.06–2.75)	0.0267
VaD^a^ (excluding AD)	9,234	127	1.78 (1.05–3.02)	0.0323
Unspecified	15,788	268	1.90 (1.29–2.80)	0.0011

Abbreviations: AD = Alzheimer disease; TBI = traumatic brain injury; VaD = vascular dementia.

The models for vascular dementia are further adjusted for ischemic heart disease and high blood pressure.

a All models are adjusted for sex, Townsend social deprivation and TBI*sex, the Charlson Comorbidity Index, and psychiatric disorders.

After excluding people with TBIs occurring within 1 year of diagnosis from this analysis, the association between TBI and AD was nonsignificant (HR = 1.44, 95% CI [0.84–2.48], *p* = 0.189), and the HR was even lower when the people with 2 diagnoses (AD and VaD) were excluded. The association between TBI and VaD showed borderline significance (HR = 1.71, 95% CI [1.06–2.75], *p* = 0.027, not significant after correction for multiple testing) and the HR increased (HR = 1.78, 95% CI [1.05–3.02], *p* = 0.032, not significant after correction for multiple testing) when the people with both diagnoses were excluded. TBI was statistically significantly associated with unspecified dementia (HR = 1.90, 95% CI [1.29–2.80], *p* = 0.0011).

## Discussion

We confirmed that history of TBI was associated with increased risk of dementia. We found that the strength of association of TBI with VaD diagnosis and TBI with unspecified dementia was higher than between TBI and AD diagnosis, although the association with VaD was not significant after correction for multiple testing. The strength of association was further increased when an individual experienced multiple TBI events and when TBI was experienced at a younger age (<60 compared with 60+). These findings were from a Welsh population–based cohort of approximately 2 million people who were alive, living in Wales, and aged between 30 and 65 years in 1999 using data from patients diagnosed with dementia during a 20-year period and controls without a dementia diagnosis at any time.

There was a high association effect size of TBI with dementia when TBI occurred within 1 year of dementia. This is likely to reflect reverse causality caused by undiagnosed dementia increasing TBI events. We also observed that the effect of TBI was increasing risk of dementia with a smaller but consistent and statistically significant effect size when TBI occurred 1–5 years or more than 5 years before dementia diagnosis.

Our finding of a link between TBI and dementia was consistent with previous population-based studies. The HR for dementia risk given a history of TBI in our study (HR = 2.32) was similar to that in previous studies (e.g., Danish study^17^ showed HR = 1.24, Swedish study^18^ reported OR = 1.81, Finnish study^16^ had estimated HR = 1.9, and Taiwanese study^29^ reported HR = 1.68). Similar to our findings, Danish^17^ and Swedish^18^ studies had shown that dementia risk was strongest in the first year after TBI, likely influenced by reverse causality, and increased with an increasing number of events. Our analyses split by the time between TBI and dementia diagnosis were less affected by reverse causality bias, and this clearly showed the consistently increased risk of dementia after TBI when the TBI occurs more than 1 year before diagnosis. Our models also included social deprivation index; models showed that the risk of dementia after TBI was lower in least deprived areas comparable with most deprived. This was consistent with previous reports.

In this study, we observed that the association between TBI and dementia was stronger between the diagnosis of both VaD and unspecified dementia compared with AD, even when adjusting for comorbidities with VaD, including stroke, hypertension, and ischemic heart disease, although this result was not statistically significant after correction for multiple comparisons. This was supported by previous findings that stroke risk was increased after TBI, with the highest risk within 4 months of TBI, but this risk is sustained for 5 years.^23^ The association between TBI and stroke was increased in older individuals compared with younger individuals.^30^ Most TBIs captured in our study involved either skull fracture or intracerebral hemorrhage and so were likely impacts of significant force causing vascular problems^31^ and are likely to lead to reduced mobility and physical activity, which can, in turn, increase the risk of stroke. Reduced mobility and physical activity are also linked to reduced cardiovascular health, which increases an individual's risk of all dementias.^32^ Furthermore, management of intracerebral bleeds after TBI can involve the temporary cessation of antiplatelet or anticoagulant medications, which in turn might increase risk of stroke and/or microvascular disease that are risk factors of VaD. The proportion of concussion in this study is low, because the data were extracted from hospital and emergency department datasets where more severe injuries are likely to be encountered. Concussion was likely under-reported because there is no objective diagnostic test; studies suggest that in suspected concussion, there are incorrect codings given in 90% of cases and concussion often presents alongside other injuries, which may receive priority when treating and reporting.^33^ However, the diagnosis of patients with dementia is challenging because of potential overlap in the clinical presentations of various dementia etiologies.^34^ Up to 84% of aged participants showed vascular pathology in addition to AD pathology.^35^ According to Ref. 36, a substantial proportion (17%) of patients with VaD are classified as misdiagnosed with AD before their confirmed VaD diagnosis. This observation could generate a spurious association of TBI with AD but not vice versa. It is challenging to speculate on why unspecified dementia diagnosis is particularly strongly associated with TBI, but it does highlight the challenge in patients receiving an accurate diagnosis. It is worth noting that the effect sizes between VaD and unspecified dementia are very similar, potentially capturing some undiagnosed VaD patients.

The novelty and strength of our study are attributed to the study design, which is based on access to a large population-based longitudinal cohort (over 20 years of clinical records), reducing issues of reverse causality and recall bias, and allowing consideration of subtypes of dementia within the same data collection framework, thus making results comparable and selection bias minimal. Data on TBI, dementia, and other diagnoses were recorded prospectively by primary and secondary care teams and did not rely on the memory of patients or their relatives, minimizing the potential for reverse causation or recall bias. We were able to account for comorbidities, including hypertension, cancers, stroke, and diabetes. Because the observation period was approximately 20 years, we were able to include individuals with TBI occurred in younger adulthood.

Our study has limitations. First, although records span over 20 years, this is too short to capture both TBIs in individuals younger than 30 years and dementia onset. Therefore, individuals who were professional/semiprofessional athletes and at the highest risk of TBI were not included, and individuals may not have reached the average age at dementia onset; therefore, it is likely that the control group would contain undiagnosed dementia cases. In addition, people assigned to the unspecified dementia group had only received an unspecified dementia diagnosis. If people were followed up for longer, they may have received a specific dementia diagnosis and would have been included in another group. This limitation may mean that older individuals included into the study with an unspecified dementia diagnosis at the beginning of the study period would have had time to be reassessed and re-diagnosed, whereas younger people at the beginning of the study may have only received an initial unspecified dementia diagnosis during the study. Second, we did not include diagnoses of TBIs registered by GPs because they are of mild severity and less reliable than those made in a hospital setting.^17^ This leads to a low overall number of people with concussion (8.4%) in our dataset, likely a large underascertainment. Future studies should aim to capture broader sources of TBI (currently unreported). Third, our data for TBI diagnosis are only available from 1999, and therefore, we might have missed some TBI exposures, which occurred in individuals at a young age. The absence of this may underestimate the association between TBI and dementia and may not reliably assess long-term consequences of concussions, which might cause problems after many years. Fourth, we may have further underestimated the risk due to overadjustment by accounting for many covariates and comorbidities. For example, we have adjusted our analyses for history of psychiatric disorders. However, because TBI was associated with both depression and dementia and psychiatric disorders are independently associated too, our results may be conservative. Fifth, we also did not consider whether standard medications/procedures for TBI treatments change risk of dementia or time to dementia or whether other medications for high blood pressure, cholesterol, or diabetes affect the association between TBI and VaD. Finally, there is a considerable overlap of vascular and AD associated pathologies and cognitive decline, and we excluded these individuals from the analyses. However, there is always a chance of misdiagnosis of neurodegenerative diseases, so the associations reported here are for reported diagnoses rather than the actual disease.

In summary, history of TBI was associated with increased risk of dementia, particularly VaD and unspecified dementia. The latter observation is novel and, therefore, requires further investigation and replication in an independent dataset. Drawing the attention of the public to TBI prevention strategies and development of novel therapeutic interventions to treat the consequences of brain injury may offer an opportunity to reduce the burden of dementia worldwide. Currently, in Europe, less than 10% of people attending an emergency department for TBI receive any follow-up; therefore, highlighting the importance of TBI treatment in the reduction of dementia risk will lead to the good practice of structured follow-up.^7^ Because TBI has chronic consequences, studies to identify components that may be more easily modified are needed. Minimizing the dementia modifiable risk factors associated with TBI such as sedentary lifestyle, poor sleep, and depression might further reduce an individual's risk of developing a neurodegenerative disease.

## Glossary

AD: Alzheimer disease
CCI: Charlson Comorbidity Index
EDDS: Emergency Department Dataset
HIV: Human Immunodeficiency Virus
HR: hazard ratio
LMIC: low-middle–income country
NHS: National Health Service
OPDW: Outpatients Database for Wales
PEDW: Patient Episode Database for Wales
SAIL: Secure Anonymised Information Linkage
TBI: traumatic brain injury
VaD: vascular dementia
WLGP: Welsh Longitudinal General Practitioner
